# ESR Essentials: common performance metrics in AI—practice recommendations by the European Society of Medical Imaging Informatics

**DOI:** 10.1007/s00330-025-11890-w

**Published:** 2025-08-03

**Authors:** Michail E. Klontzas, Kevin B. W. Groot Lipman, Tugba Akinci D’ Antonoli, Anna Andreychenko, Renato Cuocolo, Matthias Dietzel, Salvatore Gitto, Henkjan Huisman, João Santinha, Federica Vernuccio, Jacob J. Visser, Merel Huisman

**Affiliations:** 1 Artificial Intelligence and Translational Imaging (ATI) Lab, Department of Radiology, School of Medicine, University of Crete, Heraklion, Greece; 2 Computational Biomedicine Laboratory, Institute of Computer Science—Foundation for Research and Technology (ICS-FORTH), Heraklion, Greece; 3 Department of Radiology, Department of Thoracic Oncology, Netherlands Cancer Institute, Amsterdam, The Netherlands; 4 Institute of Radiology and Nuclear Medicine, Cantonal Hospital Baselland, Liestal, Switzerland; 5 ITMO University, St. Petersburg, Russia; 6 Department of Medicine, Surgery, and Dentistry, University of Salerno, Baronissi, Italy; 7 Department of Radiology, University Hospital Erlangen, Erlangen, Germany; 8 Dipartmento di Scienze Biomediche per la Salute, Università degli Studi di Milano, Milan, Italy; 9 IRCCS Istituto Ortopedico Galeazzi, Milan, Italy; 10 Diagnostic Image Analysis Group, Department of Medical Imaging, Radboud University Medical Center, Nijmegen, The Netherlands; 11 Digital Surgery LAB, Champalimaud Research, Champalimaud Foundation, Lisbon, Portugal; 12 Faculty of Medicine, Universidade de Lisboa, Lisbon, Portugal; 13 Department of Biomedicine, Neuroscience and Advanced Diagnostics (Bi.N.D.), University Hospital of Palermo, Palermo, Italy; 14 Department of Radiology & Nuclear Medicine, Erasmus University Medical Center, Rotterdam, The Netherlands; 15 Department of Radiology and Nuclear Medicine, Radboud University Medical Center, Nijmegen, The Netherlands

**Keywords:** Radiology, Artificial intelligence, ROC curve, Patient safety, Predictive value of tests

## Abstract

**Abstract:**

This article provides radiologists with practical recommendations for evaluating AI performance in radiology, ensuring alignment with clinical goals and patient safety. It outlines key performance metrics, including overlap metrics for segmentation, test-based metrics (e.g., sensitivity, specificity, and area under the receiver operating characteristic curve), and outcome-based metrics (e.g., precision, negative predictive value, F1-score, Matthews correlation coefficient, and area under the precision-recall curve).

Key recommendations emphasize local validation using independent datasets, selecting task-specific metrics, and considering deployment context to ensure real-world performance matches claimed efficacy. Common pitfalls, such as overreliance on a single metric, misinterpretation in low-prevalence settings, and failure to account for clinical workflow, are addressed with mitigation strategies. Additional guidance is provided on threshold selection, prevalence-adjusted evaluation, and AI-generated image quality assessment.

This guide equips radiologists to critically evaluate both commercially available and in-house developed AI tools, ensuring their safe and effective integration into clinical practice.

**Clinical relevance statement:**

This review provides guidance on selecting and interpreting AI performance metrics in radiology, ensuring clinically meaningful evaluation and safe deployment of AI tools. By addressing common pitfalls and promoting standardized reporting, it supports radiologists in making informed decisions, ultimately improving diagnostic accuracy and patient outcomes.

**Key Points:**

- *Radiologists must evaluate performance metrics as they reflect acceptable performance in specific datasets but do not guarantee clinical utility. Independent evaluation tailored to the clinical setting is essential*.
- *Performance metrics must align with the intended task of the AI application—segmentation, detection, or classification—and be selected based on domain knowledge and clinical context*.
- *Sensitivity, specificity, area under the ROC curve, and accuracy must be interpreted with prevalence-dependent metrics (e.g., precision, F1 score, and Matthew’s correlation coefficient) calculated for the target population to ensure safe and effective clinical use*.

## Key recommendations

- Locally validate artificial intelligence (AI) tools beyond CE-marking using datasets independent of algorithm development that reflect institutional imaging protocols and patient demographics to ensure actual performance aligns with claimed performance (Level of evidence: moderate).
- Use task-specific performance metrics such as:
  - Segmentation metrics (e.g., Dice similarity coefficient (DSC) and normalized surface distance).
  - Test-based metrics (sensitivity, specificity, area under the ROC curve) for evaluating a model’s ability to distinguish between conditions consistently across different datasets.
  - Outcome-based metrics (precision, negative predictive value (NPV), F1-score, Matthew’s correlation coefficient, area under the PR curve) for evaluating a model’s ability to make reliable diagnoses in real-world clinical settings.
  - Both test-based and outcome-based performance metrics should be assessed and reported.

(Level of evidence: moderate)

- Consider the deployment context when assessing AI performance:
  - Evaluate performance within the intended clinical setting and workflow.
  - Engage radiologists and clinicians in defining relevant metrics and estimating local disease prevalence.
  - Assess performance across clinically meaningful or vulnerable subgroups (Level of evidence: moderate).

(Level of evidence: moderate)

## Introduction

As AI becomes increasingly integrated into radiology, comprehensive evaluation of algorithm performance is crucial for safe and reliable clinical use [1]. Performance metrics of diagnostic AI must align with clinical goals, accounting for the variability of imaging modalities, scanning protocols, disease prevalence, and patient populations [1]. While traditional metrics like accuracy, sensitivity, and specificity are familiar and used most frequently, they often fail to capture real-world AI performance, especially in complex or low-prevalence scenarios [2, 3].

Performance metrics, though integral to radiological practice, are often perceived as complex. Inappropriate use of metrics is common in AI literature [4, 5], including reliance on metrics vulnerable to class imbalance, overemphasis on single metrics, and neglect of clinical relevance, which can mislead users and obscure algorithm limitations, potentially leading to overdiagnosis and increased costs [6].

Radiologists adopting AI tools are often left to interpret metrics and estimate utility without adequate guidance, risking flawed assessments. This “buyer beware” approach places the burden on end-users to evaluate workflow and patient care benefits, a time-intensive task further complicated by the lack of standardized reporting on metrics. Vendors, while emphasizing subjective benefits of their software, cannot rely solely on these alone for AI’s sustainable integration into healthcare [7].

This article, part of the “ESR Essentials” series, provides recommendations for selecting and interpreting overlap and discriminatory metrics in diagnostic AI radiology, including synthetic images, addressing pitfalls, offering mitigation strategies, and equipping radiologists to make informed decisions about diagnostic AI tools. Expert opinion on the use of these metrics has been provided in consensus by a group of experts from the European Society of Medical Imaging Informatics.

Beyond overlap and discriminatory metrics, calibration metrics (e.g., Brier score), uncertainty quantification (e.g., conformal prediction for trustworthiness), and explainability metrics (e.g., Grad-CAM) are also crucial for understanding model behavior. Brier score indicates how well the outcomes are represented by probabilistic predictions, ensuring that the predicted probabilities are well calibrated, i.e., a lower Brier score indicates that the mean square error between the probabilities and the actual outcome, is lower, and therefore the model predictions reflect the outcome well [8]. Conformal prediction is a framework for quantifying uncertainty in predictions by transforming point predictions into prediction sets. It is notable for being distribution-free, meaning it can provide valid confidence sets without assuming any specific data distribution. Such uncertainty in quantification methods assists in avoiding dangerous overconfidence in predictions that may not be correct [9]. Nonetheless, there is still limited real-world implementation of such methods in imaging applications, potentially leaving space for the overestimation of algorithm results.

## Levels of evaluation

AI models in radiology operate across different levels of input data, from pixels to patients, each requiring tailored performance metrics. At the lowest level, segmentation metrics assess the ability to label each pixel accurately. Detection metrics evaluate the model’s capacity to locate specific regions or objects, while classification assigns labels to images or lesions (e.g., benign or malignant). Prediction tasks estimate the likelihood of future events. At the patient level, AI’s real-world impact is measured in terms of clinical outcomes and healthcare efficiency.

Choosing the appropriate category for each clinical problem is crucial, often requiring combinations of metrics. For example, lung nodule AI tools must be assessed with respect to both detection and segmentation to ensure accurate localization and volumetric analysis [10].

### Segmentation, detection, and classification task evaluation

**Fig. 1:**
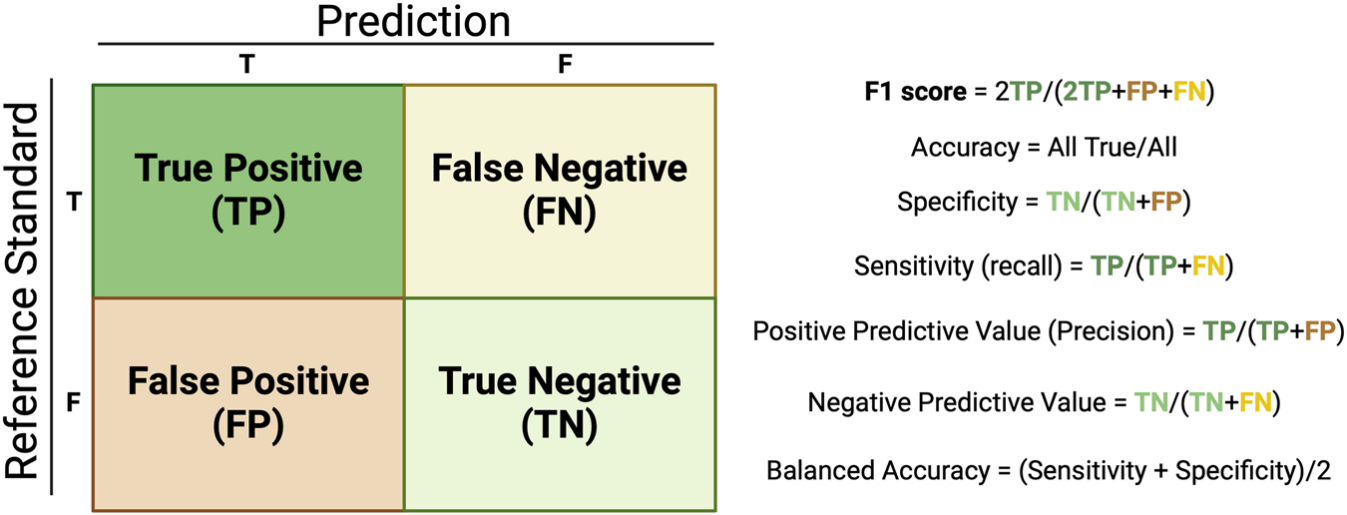
Confusion matrix. Confusion matrix example and color-coded formulas of commonly derived performance metrics (created with biorender.com)

AI algorithms can be assessed based on correct predictions (true positives [TP] and true negatives [TN]) and incorrect predictions (false positives [FP] and false negatives [FN]). The meaning of a TP varies by task level, whether at the pixel, region, scan, or patient level. The following sections outline performance metrics derived from the confusion matrix (Fig. 1) and their applications in segmentation, detection, and classification, detailing the intricacies of each task and the relevant metrics.

## Segmentation metrics

### Technical performance: segmentation

**Fig. 2:**
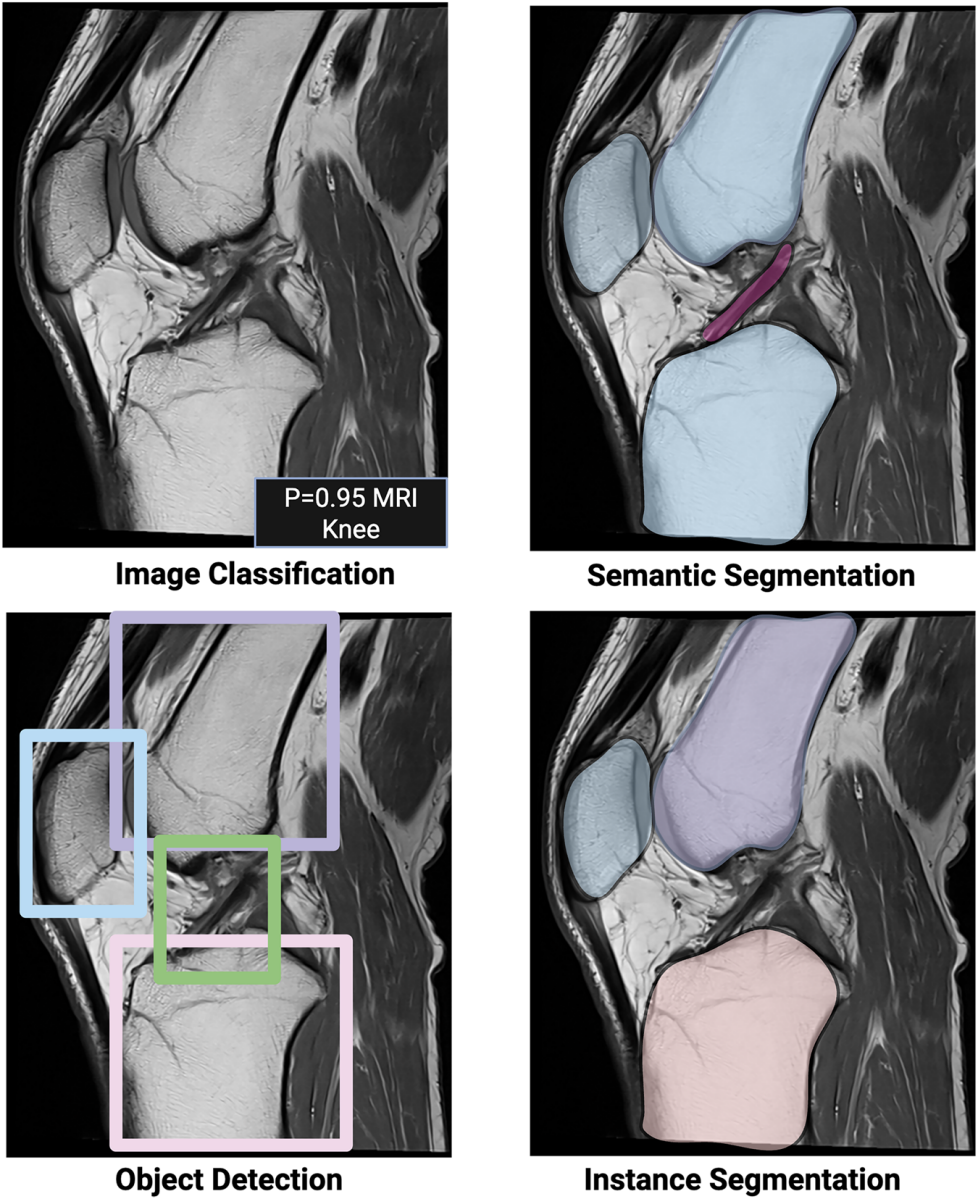
Technical performance: segmentation and object detection methods. Image classification: assigns a single probability score indicating the likelihood of the image belonging to a specific class. In this case, probability (*p*) = 0.95 for class “MRI knee” vs class “MRI hand”. Semantic segmentation: labels each pixel with a class (e.g., bones and ligaments) to create a detailed pixel-level map of the image. Object detection: identifies and localizes structures of interest with bounding boxes around anatomical regions or lesions (e.g., femur, tibia, patella, and anterior cruciate ligament) without providing segmentation. Instance segmentation: combines object detection and segmentation by labeling individual objects within the same class (e.g., the individual structures within class bones) (created with biorender.com)

**Fig. 3:**
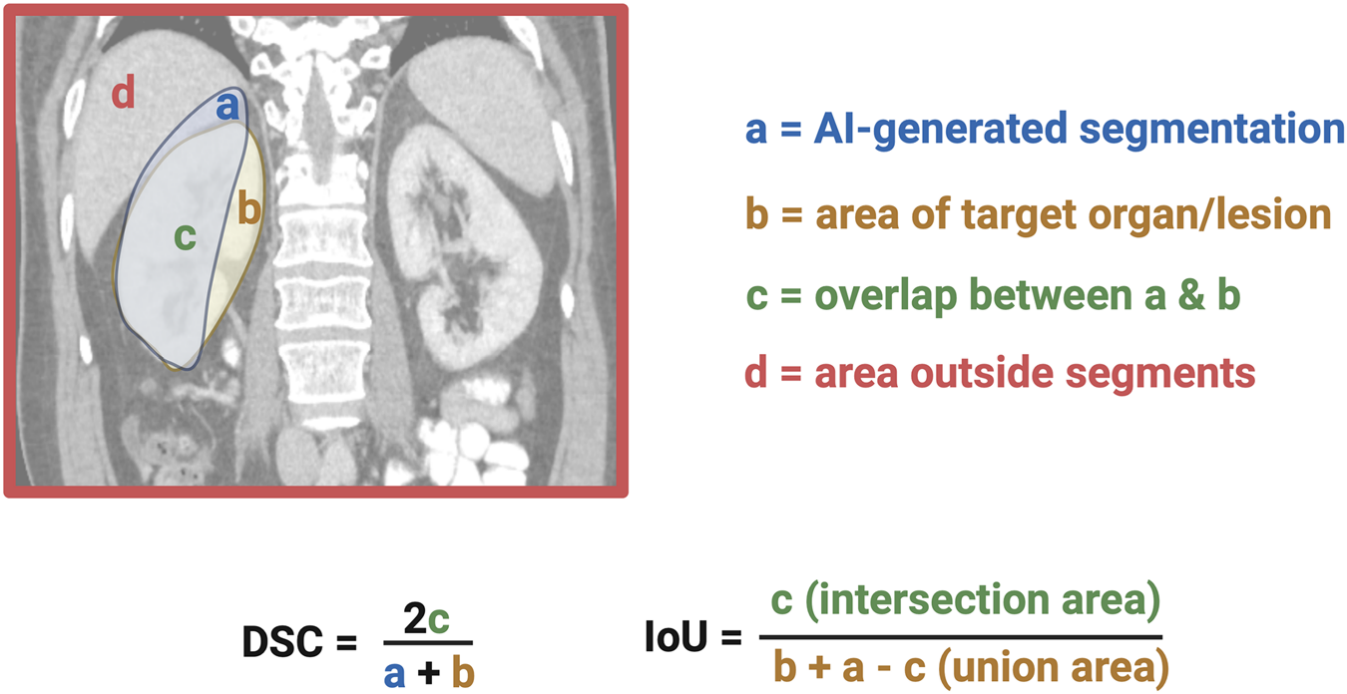
Technical performance: overlap metrics DSC and IoU. Calculation of DSC and IoU in radiology. (Exaggerated) Example of the segmentation of the right kidney on a coronal CT reconstruction image of the upper abdomen (created with biorender.com)

Segmentation tasks are divided into semantic and instance segmentation (Fig. 2). Semantic segmentation assigns each pixel to a class, while instance segmentation further distinguishes individual objects within those classes. Performance is quantified using metrics that assess spatial overlap between predicted and reference standard labels, where TP represents correctly labeled pixels. Key metrics include intersection over union (IoU), also known as the Jaccard index, and the DSC, both ranging from 0 to 1. IoU measures the ratio of overlap area to the union of predicted and actual regions, while DSC calculates twice the overlap area divided by the total number of pixels in both sets [11] (Fig. 3). Accuracy is discouraged in segmentation due to its susceptibility to pixel class imbalance, often caused by an abundance of background pixels [12].

Overlap metrics are volume-sensitive, favoring larger, spherical objects over thin or irregular structures where boundaries are more challenging. Boundary-specific metrics like the normalized surface distance and Hausdorff distance are recommended, with the first preferred for its robustness [13]. It is advisable to report DSC alongside Normalized Surface Distance in medical imaging segmentation [1]. In addition, overlap metrics are very sensitive to outliers (a single wrong pixel); thus, it is best practice to report the 99th or 95th percentile rather than the maximum distance.

### Technical performance: object detection

When pixels cluster to form distinguishable structures, object detection metrics become essential. Bounding boxes, defined by four pixel coordinates (Fig. 2), are commonly used to localize these structures. Usually, bounding boxes have only two coordinates defined (e.g., left top and right bottom). Since the bounding box is square, the other two coordinates can be calculated. In evaluating detection performance, the focus is less on precise corner placement and more on whether the predicted bounding box sufficiently matches the manual reference. This match is quantified using the IoU (see “Technical performance: segmentation”) with a bounding box considered a TP if the IoU exceeds a threshold, typically 0.1 or 0.5, depending on the clinical task. For each object class (e.g., liver and tumor), the average precision (AP) is calculated. The mean of these precision values across all classes, known as mean average precision (mAP), provides an overall detection performance score ranging from 0 to 1. Confusion matrix-based metrics can also be calculated for segmentation, which are presented in the following section.

## Classification metrics

A distinction should be made between test-based and (clinical) outcome-based performance metrics. Test-based metrics are properties of the diagnostic test or classification model itself, independent of prevalence, and allow comparison of models. Changing prevalence in the population does not affect these metrics because they only consider individuals who are known to be positive or negative. Outcome-based metrics, in contrast, depend directly on prevalence and are especially useful in clinical practice.

### Test-based (per-class) performance metrics (sensitivity and specificity)

Sensitivity (also known as recall or TP rate), is the proportion TP’s of all positives and assesses the model’s ability to correctly identify positive cases (Fig. 1). Specificity (also known as TN rate), is the proportion TN’s of all negative cases and assesses the model’s ability to correctly identify negative cases (Fig. 1). These metrics give information about the test and are prevalence independent.

### Outcome-based performance metrics (precision and NPV)

Precision, also known as positive predictive value (PPV), measures the proportion of cases predicted as positive that are truly positive (Fig. 1).

NPV measures the proportion of cases predicted as negative that are truly negative (Fig. 1). These metrics give information about the meaning of the test outcome for the patient, and are prevalence dependent.

## Importance of per-class and prediction metrics

High sensitivity and/or high PPV are essential in screening tests, depending on cultural and geographical context. High sensitivity supports ruling out conditions when combined with high NPV, but on its own, it may lead to FP, resulting in unnecessary follow-ups or interventions, but mitigate missing disease, and thereby preventing claims [2, 14]. Conversely, high specificity (low false positive rate) is vital for confirming diseases (ruling in) with severe consequences, reducing unnecessary treatments or follow-ups, particularly in invasive procedures or costly interventions (see “Low prevalence settings” section).

Sensitivity and specificity, being independent of prevalence as their formulas consider only positive or negative cases, are stable performance measures but can be misleading in settings with varying prevalence. Commonly, the prevalence of disease in studies submitted for regulatory approval is approximately 50%, primarily to avoid the need for excessively large sample sizes and the associated burden.

Precision reflects the likelihood that a positive result confirms disease in a particular patient, making it especially important in settings where FP carries significant consequences, such as in confirming malignancy (and omitting biopsy).

## Summary performance metrics

### Accuracy

Accuracy indicates the overall proportion of correct predictions (Fig. 1). Although intuitive, in class-imbalanced datasets, accuracy can appear deceptively high due to frequent prediction of the majority class. Balanced accuracy, which averages sensitivity and specificity, is a better metric in such cases as it equally weighs the detection of positives and negatives [2]. Nonetheless, balanced accuracy can still yield falsely high results in low-prevalence settings while the model is, in fact, a poor predictor. Currently, the community still tends to use accuracy even in low-prevalence scenarios, which may partially explain the commonly observed performance drop in real-life settings [5].

### F1-score

F1-score balances precision and sensitivity, which is particularly useful when there is an imbalance between sample classes where the positive class is most relevant. In other words, this metric is ideal when you do not want to miss a diagnosis while minimizing FP in a clinical setting with a relatively rare outcome.

Here, rarity is commonly defined as < 40% of patients having the disease (e.g., pulmonary nodules, incidental pulmonary embolisms, breast cancer, acute findings on brain CT, and other opportunistic screening scenarios). In these cases, the F1-score is strongly recommended over accuracy. The reason for this is that its formula does not include TNs (Fig. 1), and therefore doesn’t suffer from majority class inflation.

### Matthews correlation coefficient (MCC)

The MCC is a robust statistical metric addressing class imbalance, offering more informative and truthful scores than accuracy or even F1-score in binary scenarios [15]. It yields high values only when all four confusion matrix elements perform well, ranging from −1 (perfect misclassification) to +1 (perfect classification). Unlike F1-score, MCC remains invariant to class swapping (i.e., the positive result becoming the majority class), making it particularly valuable in imbalanced datasets [15].

Precision, NPV, accuracy, and F1-score depend on prevalence, as their formulas include both positive and negative cases. While useful for interpreting test results in specific contexts, their reliability varies across populations with differing disease prevalence. For example, an increase in prevalence raises PPV by increasing the ratio of TPs to FP. It is essential to verify whether the prevalence in a study cohort matches that of the target population, as assuming equivalence can result in significant differences between cohort-based and population-level metrics.

An example of a confusion matrix and color-coded formulas of all confusion matrix-derived metrics are presented in Fig. 1. See Pitfall 1 for a detailed example.

## Multi-threshold metrics: AUROC, AUPRC, and FROC

Multi-threshold metrics, such as the area under the receiver operating characteristic curve (AUROC) and the area under the precision-recall curve (AUPRC, or AP), summarize a model’s ability to discriminate between classes across varying thresholds. Discrimination here means the model’s ability to correctly differentiate positive cases from negative cases. Free-response operating characteristic (FROC) evaluates detection and localization performance for multiple lesions per image. AUROC and AUPRC, along with their respective curves (ROC and PR-curves), are valuable for model comparison and visualizing model behavior under specific conditions [16].

### Area under the receiver operating characteristics (ROC) curve

AUROC measures how effectively a model distinguishes positive from negative cases, indicating the likelihood that a randomly chosen positive case scores higher than a negative one. It assesses overall discrimination across all thresholds, where each threshold (operating point) represents a trade-off between sensitivity (TP rate) and specificity (TN rate). Although commonly used, a change in AUROC has little direct clinical meaning, and therefore can’t be used to assess clinical impact [17].

### AUPRC

AUPRC, or AP, quantifies the trade-off between precision and sensitivity across thresholds and is prevalence-dependent. It is particularly valuable in low-prevalence settings (see Pitfall 1 and 2). In these scenarios, AUPRC offers a far more informative real-world performance evaluation than AUROC, which can be misleadingly high due to a dominating TN class [3, 18, 19]. It can be seen as the ‘F1 score version’ of the AUROC, since both exclude the TN class.

### FROCs

FROC is used in medical imaging to evaluate diagnostic performance in tasks related to the detection and localization of multiple findings per image, such as in lung nodules, liver lesions, or metastases in general [18, 20]. As such, instead of the false-positive rate used in ROC, FROC uses false-positive per image (FPPI), which measures the average number of incorrect lesion detections per image [1]. The FROC curve plots sensitivity (on the *y*-axis) against FPPI (on the *x*-axis), showing the trade-off between correctly detecting lesions and avoiding FP. Other variations of FROC, such as localized ROC (LROC), alternative FROC (AFROC), and jackknife FROC (JAFROC), may also be used. More details about FROC alternatives can be found in references 16 and 19 [18, 21].

## Multi-class classification metrics

In cases where a model has to choose between three or more classes, appropriate evaluation metrics that reflect this complexity should be chosen. Metrics that assess performance across all classes should be used in such cases. Macro F1-score, balances both precision and recall for each class, and then averages these values, ensuring that even small classes influence the final result. On the contrary, the micro F1-score treats every prediction equally, summing up all correct and incorrect predictions before computing precision and recall, favoring larger classes. Other useful metrics include Cohen’s Kappa, which adjusts accuracy by accounting for how much agreement could happen by chance, and MCC, which captures the overall correlation between true and predicted classes, even in highly imbalanced settings [22].

### Practical application of classification metrics

In classification tasks, AI models output values (0–1 or 0–100) indicating the likelihood that an image or ROI belongs to a certain class. Actual probability can be assessed via calibration and uncertainty quantification [23]. Positive/negative assignment depends on a chosen threshold (operating point). Adjusting it aligns decisions with clinical priorities (e.g., fewer FP or negatives). For example, if the model predicts 0.6 and the threshold is 0.5, the sample is positive.

**Fig. 4:**
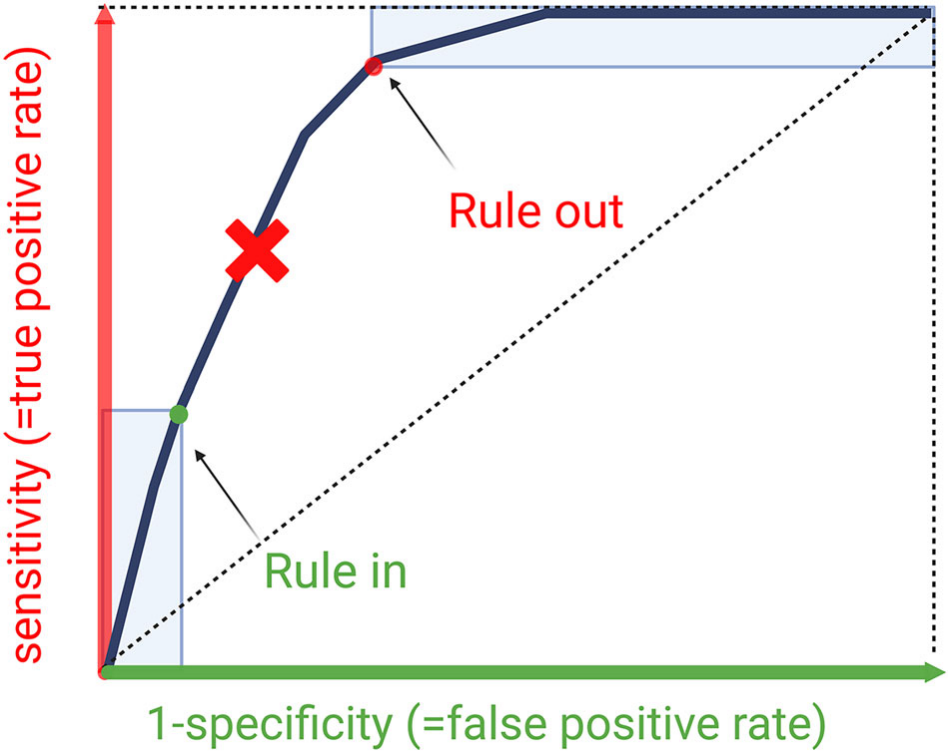
Using the ROC curve to assess the performance of an AI tool. This ROC curve illustrates the trade-off between sensitivity (TP rate) and 1-specificity (false positive rate) across various thresholds (operating points). Since AI models are not perfect, it is essential to interpret the AUC in the context of the clinical situation. In this representation, we highlight typical thresholds for a rule-in scenario and a rule-out scenario. The rule-in point prioritizes high specificity to minimize false-positive results, while the rule-out point ensures high sensitivity to avoid missing any cases. A third threshold (*X*), positioned as a theoretical optimum between these two criteria and often derived using the still-popular Youden Index, holds less clinical significance. While it aims to balance sensitivity and specificity, it often fails to meet the distinct requirements of either the rule-in or rule-out scenarios, making it less applicable in practice. While the default threshold is typically 0.5, end-users may adjust it based on clinical needs. In CE-marked tools, threshold adjustability is not always possible and should be discussed with the vendor (created with biorender.com)

For rare but serious diseases (e.g., cardiac amyloidosis and malignant glial tumors), thresholds may increase (e.g., to 0.7) to emphasize specificity, sacrificing some sensitivity and rule-in disease. This adjustment corresponds to a shift to a lower left operating point on the ROC curve or a higher left operating point on the PR curve, with each threshold generating a unique (theoretical) confusion matrix (Fig. 4). For example, with a 0.7 threshold, 0.6 becomes negative.

Traditionally, the Youden Index is used to derive sensitivity and specificity from the ROC curve. However, we advise against using the Youden Index uncritically, as it weights sensitivity and specificity equally—an approach that is rarely meaningful in clinical practice. Instead, thresholds should be interpreted and determined specifically within the context of the diagnostic window (Fig. 4). Another method is selecting minimally required sensitivity/NPV or specificity/precision according to clinical needs. For example, in cases of brain tumors, where the goal is to avoid unnecessary biopsy and/or unnecessary brain surgery, a false positive can have more severe consequences than an FN. In these cases, PPV and specificity are more important, and an operating point more towards the left of the ROC curve is chosen.

It is important to note that the predicted probabilities are not necessarily indicative of the true likelihood of a diagnosis unless the model is well calibrated. Therefore, threshold adjustment should be considered when applying the model to new data to account for potential discrepancies such as high-probability FP or low-probability FNs.

### Assessment of AI-generated images

With the rise of generative AI, synthetic medical images are increasingly used by MRI and CT vendors for AI-assisted reconstruction and for dataset augmentation in model development. Metrics commonly used to evaluate these images include the structural similarity index measure (SSIM), peak signal-to-noise ratio (PSNR), and root mean square error (RMSE). RMSE represents the square root of the cumulative squared error between the AI-generated and original images, with lower values indicating better quality [24]. PSNR measures peak signal relative to noise, where higher values signify improved quality [25]. SSIM, ranging from −1 to 1, evaluates luminance, contrast, and structure, with 1 indicating perfect similarity. Higher SSIM values reflect better perceptual quality and resemblance to the original image [26]. Of note, these metrics do not necessarily reflect diagnostic quality. Therefore, an image with low SSIM, low PSNR, and high RMSE can still have high diagnostic quality if the key finding is clear. In addition, SSIM, often misunderstood, reflects overall similarity, meaning images with blurred edges may score lower even if the diagnostic area is intact. Therefore, these metrics should always be accompanied by human evaluation of diagnostic quality and interchangeability [27, 28].

## Metrics for the evaluation of clinical trials with AI

The design and execution of clinical trials (i.e., prospective studies) are of utmost importance for the incorporation of AI in clinical workflows. In prospective clinical studies, outcomes differ compared to (retrospective) diagnostic studies. Clinical (i.e., patient or hospital level) endpoints will serve as primary metrics to assess the clinical performance of AI models, complemented with traditional diagnostic performance metrics. Such endpoints include recall rate and interval cancer rate (in breast cancer screening), mortality, hospitalization rates [29], length of hospital stay]^30^], acute hospital bed days [31], waiting times for treatment [32], disease-free survival [33], patient satisfaction rates [34]. Data on some endpoints, like interval cancer rate, may take years to collect. At the moment, limited medical imaging trials with AI have been performed, partially due to AI being a relatively young field, but their number is expected to rise in the near future.

## Pitfalls in the use of metrics and mitigation strategies

**Fig. 5:**
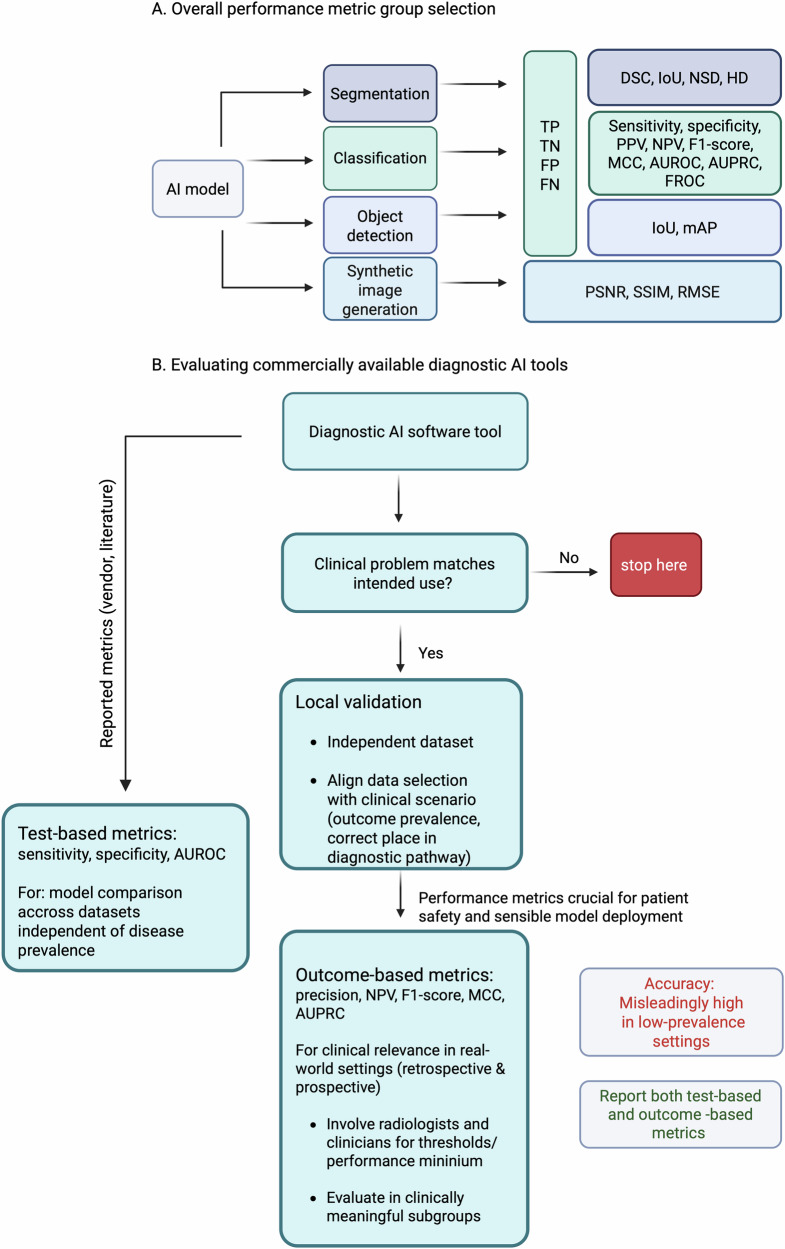
Flowchart depicting the strategy for selection of appropriate performance metrics per AI model task (**A**). Flowchart depicting the evaluation of commercially available diagnostic (classification) AI tools (**B**)

A series of these common pitfalls in current AI in medical imaging are described below, with an example of the most common clinical pitfall. Mitigation strategies are provided in Table 1. For a comprehensive list of common and rare pitfalls in the use of metrics, not limited to radiology, readers can refer to the Metrics Reloaded online tool (https://metrics-reloaded.dkfz.de/). A Flowchart (Fig. 5) has been provided that depicts the strategy for the selection of appropriate performance metrics per AI model task and the strategy for the evaluation of commercially available diagnostic AI tools.

**Table 1:** Summary of common pitfalls and mitigation strategies

Pitfall	Mitigation strategy
Segmentation metrics	
1. Overlooking small structures	• Report multiple metrics, such as DSC, to evaluate overall volumetric overlap and NSD as an evaluation of the boundary of the segmentation. For multiple non-connected objects in the segmentation task, report detection metrics as well, based on IoU > 0.1, for example [1]. • Consider weighted metrics that give more importance to critical regions (e.g., normalized surface distance for boundaries and centerline-DSC for tubular structures). • Evaluate performance separately for different anatomical structures or lesion sizes (e.g., plot the volume on the *x*-axis and DSC on the *y*-axis).
2. Shape unawareness	• Use a normalized surface distance with different values of cutoffs based on the granularity of the structure and voxel spacing (e.g., NSD at 1 mm, NSD at 3 mm, etc.). • Visually inspect results to complement quantitative metrics.
Classification metrics	
3. Overreliance on single metrics	• Sensitivity and specificity should always be interpreted in conjunction with precision and other prevalence-dependent metrics (e.g., F1 score, MCC, and false discovery rate), calculated using the target population’s prevalence, to ensure safe and efficient clinical use. This can be done by using an online calculator (e.g., https://www.medcalc.org/calc/diagnostic_test.php (changeable prevalence) or https://www.omnicalculator.com/statistics/confusion-matrix (includes F1 score and MCC; adjust prevalence yourself in the confusion matrix cells (also see the example in Fig. 6B)) [37]. • “One metric is no metric”. Different metrics capture different aspects of the model and should always be used in conjunction with each other.
4. Relying on accuracy for real-world performance	• In low-prevalence settings, the F1-score and MCC are preferred over accuracy as a summary metric. • Precision-recall curves, which exclude TNs, are particularly useful for evaluating model performance in highly imbalanced datasets, as performance inflation by the majority class is avoided.
5. Ignoring clinical context	• Assess algorithm performance under the prism of its specific clinical application with associated metrics. • Involve radiologists and clinicians in estimating local disease prevalence, defining relevant performance metrics, and selecting operating points for performance assessment [38]. • Evaluate performance in clinically meaningful subgroups [39, 40]. • Ensure that metrics and impact/value also apply to the local situation where a model is intended to be used [41].
Reporting	
6. Inadequate Reporting	• Follow reporting guidelines like CLAIM for AI in medical imaging [42, 43] and CLEAR for radiomics research [44], uploading the completed checklist together with the manuscript. • Clearly describe the evaluation dataset, including study sample baseline, confounding comorbidities, and outcome characteristics [45] • Report both summary metrics and performance breakdowns for relevant subgroups, as well as the confusion matrix for the threshold of the main outcome. • Statistically compare algorithm performance (e.g., compare AUROCs with DeLong’s test) and report *p*-values together with confidence intervals [46].

### Pitfall 1. Overlooking small structures

Large ROIs may fail to capture small but clinically important structures. Known as small size bias, this issue is akin to class imbalance, where small structures (e.g., small areas within a large ROI) contribute less to overall metrics, including sensitivity and DSC.

### Pitfall 2. Shape unawareness

Pitfall: Some metrics, like DSC, may not fully capture shape differences in segmentation tasks.

### Pitfall 3. Low-prevalence settings in combination with overreliance on single metrics and regulatory approval

Low prevalence settings can either occur in rare diseases in secondary referral centers, or low occurrence of common conditions, such as in screening, defensive medical cultures, or tertiary referral centers.

**Fig. 6:**
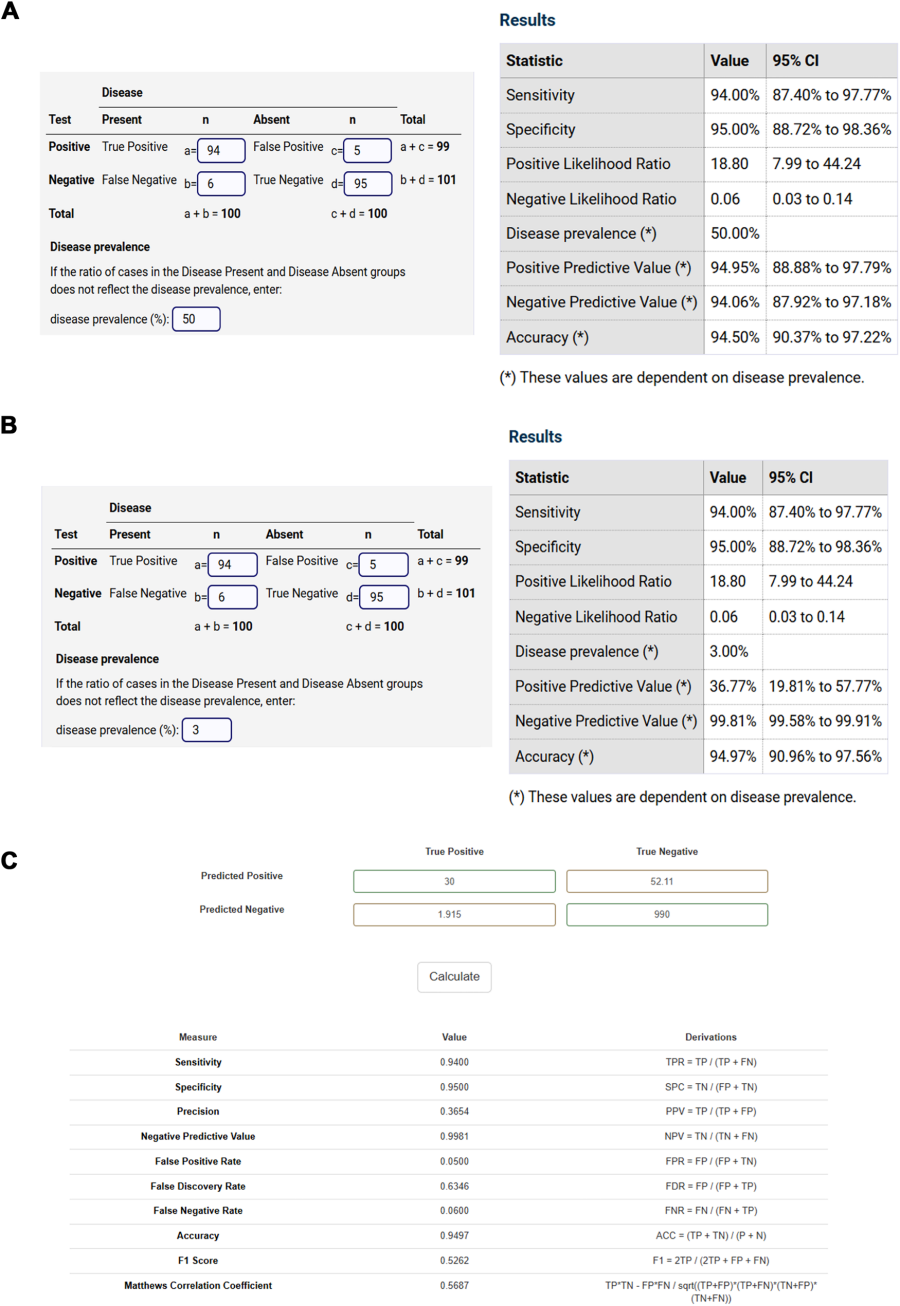
Example of a confusion matrix with associated prevalence-dependent prediction metrics of a pulmonary embolism (IPE) detection algorithm with a sensitivity of 94% and a specificity of 95% (Images have been obtained from MedCalc Software Ltd. Diagnostic test evaluation calculator. https://www.medcalc.org/calc/diagnostic_test.php (version 23.0.9; accessed December 11, 2024)). **A** Results with an IPE prevalence of 50% (possible theoretical performance) × confidence interval (95% CI) width depends on sample size. **B** Results with an IPE prevalence of 3% (possible ‘real-world’ performance) × confidence interval (95% CI) width depend on sample size. **C** F1 score and MCC in with an IPE self-set prevalence of 3% (possible ‘real-world’ performance)

The most common scenario currently encountered in clinical practice is that a seemingly small flaw in specificity can result in a disproportionately large number of FP in low-prevalence settings. For instance, a CE-marked and FDA-cleared incidental pulmonary embolism detection algorithm with a sensitivity of 94% and a specificity of 95% will have an accuracy of 95%, and a false positive rate of 5%, in a center with 3% prevalence of IPE, which sounds adequate at first sight [35]. However, out of all flagged cases, most (63%) will be false alarms (false discovery rate = 1 − precision), potentially leading to even more overwhelmed clinical workflows with extra scans and possibly overtreatment with anticoagulants (Fig. 6). This is especially worrisome as radiologists are likely to follow AI results [36]. Also, it may very well be that actual performance is even worse due to data drift and heterogeneous human-machine interaction.

If the F1-score (53%), MCC (57%), and precision (37%) had been available upfront (Fig. 6B), it would have been clear that deploying this algorithm was not advisable. Of note, vendors often expect end-users to perform these evaluations themselves. The fact that an algorithm has obtained a CE mark implies safety, it does NOT imply clinically meaningful use.

### Pitfall 4. Relying on accuracy for real-world performance

Relying on accuracy can be misleading for imbalanced datasets, as a model could achieve high accuracy by simply predicting the majority class, most commonly TNs (also see Fig. 6B, C).

### Pitfall 5. Ignoring clinical context

Focusing solely on statistical performance without considering clinical utility.

### Pitfall 6. Inadequate reporting

Appropriate reporting of performance metrics is of utmost importance for the completeness of an AI manuscript. Insufficient reporting is common in the relevant literature.

## Summary statement

In summary, this paper underscores the importance of robust evaluation metrics for safely integrating AI into radiology. Practical strategies address pitfalls such as reliance on single metrics and poor reporting, equipping radiologists to critically appraise AI tools for safe clinical use and vendors and researchers for more transparent reporting.

## Patient summary

AI is used more and more in radiology to detect and diagnose diseases faster and more accurately. Evaluation of the performance of these tools is important not only in research but also in real-world settings in hospitals, to ensure safety and accuracy. This guide helps radiologists choose the right ways to measure AI performance, aiming to keep patients safe and maintain high diagnostic quality.

## Abbreviations

AP: Average precision
AUPRC: Area under the precision-recall curve
DSC: Dice similarity coefficient
FN: False negatives
FP: False positives
FROC: Free-response operating characteristic
IoU: Intersection over union
MCC: Matthews correlation coefficient
NPV: Negative predictive value
PPV: Positive predictive value
PSNR: Peak signal-to-noise ratio
RMSE: Root mean square error
SSIM: Structural similarity index measure
TN: True negative
TP: True positive
